# S1 Brain Connectivity in Carpal Tunnel Syndrome Underlies Median Nerve and Functional Improvement Following Electro-Acupuncture

**DOI:** 10.3389/fneur.2021.754670

**Published:** 2021-10-27

**Authors:** Harrison Fisher, Roberta Sclocco, Yumi Maeda, Jieun Kim, Cristina Malatesta, Jessica Gerber, Joseph Audette, Norman Kettner, Vitaly Napadow

**Affiliations:** ^1^Department of Radiology, Athinoula A. Martinos Center for Biomedical Imaging, Harvard Medical School, Massachusetts General Hospital, Boston, MA, United States; ^2^Department of Radiology, Logan University, Chesterfield, MO, United States; ^3^Department of Physical Medicine and Rehabilitation, Spaulding Rehabilitation Hospital, Boston, MA, United States; ^4^Division of Clinical Medicine, Korea Institute of Oriental Medicine, Daejeon, South Korea; ^5^Department of Pain Medicine, Harvard Vanguard Medical Associates, Atrium Health, Boston, MA, United States

**Keywords:** carpal tunnel syndrome, electro-acupuncture, functional magnetic resonance imaging, functional connectivity, peripheral nerve function

## Abstract

Carpal Tunnel Syndrome (CTS) is a median nerve entrapment neuropathy that alters primary somatosensory cortex (S1) organization. While electro-acupuncture (EA), a form of peripheral neuromodulation, has been shown to improve clinical and neurophysiological CTS outcomes, the role of EA-evoked brain response during therapy (within and beyond S1) for improved outcomes is unknown. We investigated S1-associated whole brain fMRI connectivity during both a resting and sustained EA stimulation state in age-matched healthy controls (*N* = 28) and CTS patients (*N* = 64), at baseline and after 8 weeks of acupuncture therapy (local, distal, or sham EA). Compared to healthy controls, CTS patients at baseline showed decreased resting state functional connectivity between S1 and thalamic pulvinar nucleus. Increases in S1/pulvinar connectivity strength following verum EA therapy (combined local and distal) were correlated with improvements in median nerve velocity (*r* = 0.38, *p* = 0.035). During sustained local EA, compared to healthy controls, CTS patients demonstrated increased functional connectivity between S1 and anterior hippocampus (aHipp). Following 8 weeks of local EA therapy, S1/aHipp connectivity significantly decreased and greater decrease was associated with improvement in patients' functional status (*r* = 0.64, *p* = 0.01) and increased median nerve velocity (*r* = −0.62, *p* = 0.013). Thus, connectivity between S1 and other brain areas is also disrupted in CTS patients and may be improved following EA therapy. Furthermore, stimulus-evoked fMRI connectivity adds therapy-specific, mechanistic insight to more common resting state connectivity approaches. Specifically, local EA modulates S1 connectivity to sensory and affective processing regions, linked to patient function and median nerve health.

## Introduction

Carpal tunnel syndrome (CTS) is an entrapment neuropathy of the median nerve characterized by sensations of pain and paresthesia and reduced velocity (or increased latency) for impulses traveling over the affected median nerve. Although CTS is a peripheral neuropathy, our prior studies have also identified structural and functional alterations in the central nervous system. In the brain, gray matter plasticity in primary somatosensory (S1) and motor (M1) areas has been associated with altered median nerve conduction (1) and sensations of pain and paresthesia (2). CTS patients also show altered white matter integrity within S1 (3). Functional neuroimaging has revealed altered S1 somatotopy, including reduced separation between cortical representations of the second (D2) and third (D3) digits of the hand (4, 5). In particular, the distance between D2 and D3 and that of D2 and D5 are decreased while the distance between D3 and D5 remains unchanged, suggesting that the representation of D2 is shifted dorsally toward D3. However, the role of other cortical areas, and even subcortical brain regions in CTS pathology has been less studied.

While severe median nerve compression in CTS is often treated surgically, conservative management with non-surgical and non-pharmacological interventions also has therapeutic benefit (6). Acupuncture, particularly electro-acupuncture (EA), a hybrid of traditional and modern neuromodulation techniques (7), was shown to improve maladaptive neuroplasticity, as well as median nerve conduction and CTS symptom severity (3). A study of evoked brain fMRI response found that EA at the hand/wrist produced contralateral S1 activation in the hand/wrist representation area which was linked with both median nerve latency (8) and post-stimulation reduction in CTS pain and paresthesia (9). Furthermore, a longitudinal course of EA therapy applied either locally (i.e., hand/wrist area) or distally (i.e., leg/ankle area) reduced CTS symptoms and improved both peripheral nerve function and central maladaptive neuroplasticity within S1 (3), suggesting the involvement of brain-based mechanisms of action. However, central regulation of symptom severity and peripheral nerve health likely involves brain regions outside of S1 and the role of other cortical/subcortical areas in the beneficial clinical effects of local and distal EA is unknown.

Functional connectivity analyses have assessed cross-regional communication via analysis of spontaneous fluctuations of activity across the brain, most commonly in the absence of a task (10). However, similar “resting” connectivity analyses can also be performed with fMRI data collected during sustained stimuli, e.g., ranging from cuff pressure (11, 12) to cutaneous electrostimulation (13). The latter, in particular, is similar to EA, and sustained-EA connectivity analyses could potentially uncover brain-based mechanisms of action relevant to a longitudinal course of EA therapy in CTS. Given that acupuncture (and also EA) is a somatosensory-based neuromodulatory technique (14), we chose S1, specifically the D2 representation (4), as the seed for our investigations of resting state and sustained-EA functional connectivity. We hypothesized that (1) CTS-associated functional plasticity extends beyond S1 somatotopy to include resting S1 connectivity between cortical representations for affected digits and other brain regions, (2) S1 connectivity during EA differs between CTS and healthy controls, and (3) longitudinal distal and local EA therapy alter functional connectivity patterns linked to CTS pathology and clinical outcomes.

## Methods

This study was conducted as a single-center, blinded, placebo controlled, randomized parallel-group longitudinal neuroimaging study, pre-registered with ClinicalTrials.gov (NCT01345994). The study took place at Spaulding Rehabilitation Hospital (SRH) and Athinoula A. Martinos Center for Biomedical Imaging, Department of Radiology, Massachusetts General Hospital (MGH), in Boston, MA from January 2009 to December 2014. All study protocols were approved by MGH and Partners Human Research Committee and all subjects provided written informed consent. Further results of clinical and neuroimaging data not included in this particular analysis and further methodological detail are also available in our previous publication (3).

### Participants

Patients diagnosed with CTS, 20–65 years old, were recruited and consented, followed by baseline evaluation by study physician (blinded to allocation), who captured symptom history and conducted physical examination using Phalen's maneuver (15) and Durkan's sign (16). Subjects then underwent nerve conduction studies (Cadwell Sierra EMG/NCS Device) for median and ulnar, sensory and motor nerve conduction values for both hands. Inclusion criteria for mild/moderate CTS required a history of pain/paresthesia in median nerve innervated territories for >3 months duration, median sensory nerve conduction latency > 3.7 ms for mild CTS, >4.2 ms for moderate CTS, and/or >0.5 ms compared to ulnar sensory nerve latency for both mild and moderate CTS, with normal motor conductions. Subjects with >4.2 ms median nerve motor latency and >50% loss of motor amplitudes were considered to be “severe” CTS and were excluded from this study. For subjects diagnosed with bilateral CTS, clinical variables from the reported more affected hand were used for primary outcomes. Exclusion criteria were as follows: contraindications to MRI, history of diabetes mellitus, rheumatoid arthritis, wrist fracture with direct trauma to median nerve, current usage of prescriptive opioid medication, severe thenar atrophy, previous acupuncture treatment for CTS, non-median nerve entrapment, cervical radiculopathy or myelopathy, generalized peripheral neuropathy, severe cardiovascular, respiratory, or neurological illnesses, blood dyscrasia or coagulopathy or current use of anticoagulation therapy. Healthy control subjects, 20–65 years old, with identical exclusion criteria, were also recruited for baseline neuroimaging comparisons.

### Study Protocol

Following baseline clinical assessment, eligible CTS subjects were randomly assigned to one of three parallel study arms. We used computer generated permuted block randomization (blocks of six), stratified by CTS severity (mild/moderate). For the clinical intervention, the acupuncturist was informed of group allocation at the first treatment visit. The three intervention arms were (i) verum acupuncture “local” to the more affected hand; (ii) verum acupuncture at “distal” body sites, contralesional to the more affected hand; and (iii) sham acupuncture using non-penetrating placebo needles. Brain MRI scans were obtained at baseline and post-therapy. Nerve conduction studies were also obtained at baseline and post-therapy while symptom severity, assessed with the Boston Carpal Tunnel Syndrome Questionnaire (BCTQ) (17), was additionally assessed at 3-month follow-up. MRI assessments were conducted at baseline and following acupuncture therapy and included fMRI data collection during a resting state and during a sustained electro-acupuncture stimulation state according to group allocation.

The acupuncture therapy protocol has been previously described (3). Briefly, sixteen treatments were delivered over an 8-week period in a tapered schedule (three times, two times, and then once per week). Local acupuncture treatment was administered at two locations on the most-affected lower arm (TE5, PC7) which were stimulated by electro-acupuncture, standardized across all subjects in this group, and three additional lower arm locations selected by the clinician out of six possible locations (HT3, PC3, SI4, LI5, LI10, and LU5), stimulated with manual acupuncture. Distal acupuncture treatment was administered to two locations on the lower leg opposite to the most-affected hand (SP6, LR4) which were stimulated by electro-acupuncture, with three additional manual acupuncture points (GB34, KI3, and SP5) all on the lower leg. For the sham group, non-insertive placebo needles were placed over the skin at five non-acupuncture points on the lower arm, with electrodes attached to two placebo needles, but with no electrical current passed. Subjects were told that they “may or may not feel electrical sensations” during the therapy. Electro-acupuncture was delivered during fMRI as well, according to group allocation (see below).

### Clinical Outcomes

The BCTQ (17) was administered at baseline, post-therapy, and at 3-month follow-up. Patients self-rate items on a 1–5 scale and items are aggregated into a symptom severity scale, which includes 10 items regarding pain and numbness severity, and an 8-item Function Severity Scale, which assesses precise hand movement tasks. Nerve conduction studies were performed according to previously described standard methods (8, 18), at baseline and post-therapy. Median sensory nerve conduction velocity was calculated from the average of digit 2 (D2) and digit 3 (D3) measurements from the more affected hand. Baseline differences between HC and CTS subjects were assessed for BCTQ and nerve conduction velocity with unpaired *t*-tests. Repeated measures ANOVA models were used to compare post-therapy and baseline values across treatment groups in the CTS cohort.

### Resting-State and Sustained Electro-Acupuncture fMRI

In order to assist with fMRI data normalization, structural brain MRI data were acquired with a multiecho MPRAGE T1-weighted pulse sequence (TR 5 2,530 ms, TE1/TE2 = 1.64/30.0 ms, TI = 1,200 ms, flip angle = 7°, field of view = 256 × 256, slices = 176, sagittal acquisition, spatial resolution = 1 × 1 × 1 mm^3^) on a 3T Siemens Trio scanner (Siemens Medical, Erlangen, Germany) equipped with a 32-channel head coil.

Functional MRI data were acquired using a gradient echo blood oxygen level-dependent (BOLD) T2^*^-weighted echo-planar imaging (EPI) pulse sequence (repetition time/echo time = 2,000/30 ms, 32 coronal slices, voxel size = 3.125 × 3.125 × 3.0 mm^3^, volumes = 180) with Prospective Acquisition CorrEction (PACE). We collected BOLD fMRI data during a 6-min resting state during which subjects were instructed to keep their eyes open. Acupuncture needles were then placed according to participant treatment group for electro-acupuncture. As in the clinical acupuncture protocol, subjects randomized to the local acupuncture group had needles inserted at TE5 and PC7 at the more affected forearm, for the distal group at LR4 and SP6 on the opposite leg, and for the sham group at two non-acupuncture points on the more affected forearm (3, 8). For verum EA, MRI-compatible titanium needles (0.2 mm in diameter, 35–50 mm in length, DongBang Acupuncture Inc. Boryeong, Korea) were inserted and deqi sensation elicited. For sham EA, MRI-compatible blunt-tipped acupuncture needles were placed with a single tap but not inserted percutaneously, over sham points, SH1 and SH2. For all 3 groups, needles were connected to a constant current EA device (HANS LH202H, Neuroscience Research Center, Peking University, Beijing, China). A licensed acupuncturist trained to place and stimulate acupuncture needles in the scanner performed these procedures.

Two 6-min BOLD fMRI scans were collected during which electro-acupuncture was continuously delivered at 2 Hz, recalibrating stimulation intensity as needed between scans to maintain a perceived intensity of moderately strong but not painful for the active stimulation group subjects. Participants were instructed to focus their attention on the electro-acupuncture needles, including those in the Sham group even though no electrical current was delivered for this group. Subjects in this group were instructed that the current intensity was set to a predetermined level and that they may or may not feel any sensation at the needle sites. Healthy controls attended three separate imaging visits, receiving either Local, Distal, or Sham electro-acupuncture in a randomized order, with similar procedures as above.

### BOLD fMRI Pre-processing

BOLD fMRI data were first corrected for noise associated with cardiac and respiratory pulsatility [3dretroicor, AFNI, (19)]. As a whole, 87% of the BOLD fMRI data had corresponding physiological data of sufficient quality to perform the correction. Data were then preprocessed with FMRIPREP [version 1.5.8, (20)] which was used to segment the anatomical MRI volume into constituent tissues (FSL fast), realign BOLD fMRI volumes shifted by head motion [mcflirt, FSL 5.0.9, (21)], and perform co-registration of anatomical and BOLD images to standard space [MNI 2009c Asymmetrical template, (22)]. More specifically, FMRIPREP uses boundary-based registration [bbregister, FreeSurfer, (23)] to register the realigned BOLD fMRI data to the anatomical MRI volume. Spatial normalization of the anatomical MRI volume to standard space was performed through a non-linear registration [antsRegistration, ANTs 2.2.0, (24)]. All transformations above (realignment, co-registrations) were combined into a single interpolation step (ApplyTransforms, ANTs). Physiological and scanner-related noise was estimated from non-parenchyma tissue [aCompCor, (25)], which complements the RETROICOR algorithm by also targeting additional sources of artifacts beyond those introduced by cardiac and respiratory signals. Noise regressors were generated from principal components estimated from high-pass filtered (discrete cosine filter with 128 s cut-off) preprocessed BOLD fMRI signals in eroded white matter and cerebrospinal fluid masks in participants' anatomical MRI volume. Following FMRIPREP, BOLD fMRI data were skull stripped (3dSkullStrip, AFNI). The noise regressors were removed using a general linear model (GLM) that contained six head motion realignment parameters (translations and rotations), the top six CompCor components, a censoring confound matrix of high head motion time points (framewise displacement > 0.7 mm) (26), a temporal bandpass filter (0.008–0.1 Hz), and Gaussian spatial smoothing (full width half maximum 5 mm). This GLM was applied as a single step (3dTproject, AFNI), performing the complete nuisance regression and temporal filtering simultaneously to avoid reintroducing confounds into the analysis, which can be a problem with modular preprocessing steps (27). Global signal was not included in this step. The spatial smoothing is performed by 3dTproject as a second step after the timeseries regression is complete. The smoothed residual signal was then used for functional connectivity analyses. BOLD data that had excessive motion (>1 mm mean framewise displacement, or more than thirty timepoints exceeding 0.7 mm of framewise displacement) were dropped from analyses. Detailed reporting on the number of scans that passed quality control for use in functional connectivity analyses is found in Supplementary Table 1. Comparisons between CTS and healthy control groups used the resting BOLD fMRI data from healthy controls' first MRI visit only (*N* = 28), in order to match for novelty effects between baseline CTS and healthy control groups.

### Resting State and Sustained Electro-Acupuncture Seed-to-Voxel Functional Connectivity Analyses

Seed-to-voxel connectivity maps were used to assess whole brain connectivity to the CTS-affected S1 hand region. We used a S1 D2 finger seed (3 mm radius spherical ROI, MNI coordinates: ±51, −18, 54) located in Broadman Area 1 (known to exhibit somatotopic differentiation), defined by the peak S1 activation during somatosensory stimulation in an independent cohort of CTS patients (4). A 3 mm radius was chosen for the D2 seed as a compromise between somatotopic specificity and generalizability given the shifts in individual functional representations of D2 with EA therapy (3).

Importantly, the seed region was placed contralateral to the more affected hand, or contralateral to the dominant hand for healthy controls. However, the whole brain BOLD fMRI data were *not* flipped for connectivity analyses in order to maintain any lateralized functional brain specificity across right- and left-lesioned CTS patients. Functional connectivity maps were generated (fsl_glm, FSL) with the variance normalized seed timeseries as the regressor of interest. Seed-to-voxel connectivity maps for first and second EA scan runs were combined at the subject level with a fixed effect model. Group differences in connectivity were assessed at baseline using mixed effect models [FSL expert analysis tool (FEAT), FSL] contrasting CTS patients and HC for REST and for EA separately, specific to EA group allocation. All seed-to-voxel group results were corrected for familywise error with a cluster-forming threshold of 2.3 and corrected cluster size threshold of *p* < 0.05. This choice of thresholding was based on previous work demonstrating that a cluster forming threshold of 3.1 in FSL FLAME was overly conservative for two sample *t*-tests, whereas 2.3 more appropriately generated family wise error rates of ~5% in null data (28). An additional analysis was performed on functional connectivity between somatotopic subregions of S1, using seeds contralateral and ipsilateral to the affected hand (Supplementary Materials).

### Region of Interest Analyses

Follow-up region of interest (ROI) analyses were used to evaluate the association between brain connectivity and clinical outcomes (e.g., nerve conduction velocity, BCTQ scores) as well as how acupuncture therapy modulated brain connectivity. ROIs were selected from significant clusters in the REST and EA baseline CTS vs. HC difference maps. Functional connectivity values were extracted from 3 mm radius spheres centered on the peak voxel from significant clusters. For the ROIs extracted from each of local, distal, and sham EA baseline group comparisons, a one-way ANOVA was used to compare D2 ROI connectivity values across these EA types within the CTS cohort. Additionally, unpaired *t*-tests were used to see if the CTS vs. HC group difference was present for local, distal, and sham EA types as well. Post-therapy changes in REST and EA connectivity values were assessed by repeated measures ANOVA models (rstatix, R, https://www.R-project.org/) with factors Treatment (local, distal, and sham), and Time (baseline and post-therapy). *Post-hoc t*-tests were performed to further explore how longitudinal therapy impacted ROI-to-ROI connectivity. Specifically, these analyses also correlated post-therapy changes in D2 ROI connectivity values during REST and EA with post-therapy changes in nerve conduction velocity and BCTQ function scores for each therapy group.

Follow-up analyses were also performed to verify that differences between left and right hand affected CTS patients did not affect the results of the seed to voxel functional connectivity analyses (Supplementary Materials).

## Results

### Demographic and Clinical Characterization

After processing the BOLD fMRI data and controlling for data quality, twenty-eight (*N* = 28) healthy controls and sixty-four (*N* = 64) CTS patients contributed BOLD fMRI data at baseline. Forty-seven (*N* = 47) of these CTS patients contributed post-therapy BOLD fMRI data. No significant differences in age were found between CTS and HC groups, as well as between the acupuncture therapy groups within the CTS cohort (Supplementary Table 2). While all HC subjects were right handed, 64% (right hand CTS: *N* = 41, left hand CTS: *N* = 23) of the CTS subjects were more affected in their right hand. Proportions affected hand did not differ between treatment groups for CTS patients [χ^2^ (df = 2, *N* = 65) = 0.93, *p* = 0.63, Supplementary Table 2]. As expected, in comparison to healthy controls, CTS patients had lower median nerve velocity (unpaired *t*-test CTS: 38.00 ± 7.59 m/s, HC: 53.61 ± 5.50 m/s, *p* < 0.001). Following acupuncture therapy, nerve conduction velocity significantly increased in the local + distal (combined verum) group only (paired *t*-tests, local (*N* = 20): 0.67 ± 2.66 m/s, *p* = 0.28, distal (*N* = 18): 1.24 ± 2.78 m/s, *p* = 0.074, sham (*N* = 21): −0.38 ± 3.57 m/s, *p* = 0.63, verum (*N* = 38): 0.94 ± 2.69 m/s, *p* = 0.038) (Supplementary Table 3). The BCTQ function scores showed a significant effect of Time (baseline, post therapy, 3 month follow-up, *F* = 13.8, *p* < 0.001). Local, sham, and verum acupuncture groups showed significantly reduced (i.e., improved) scores and distal group improvements also trended toward significance [paired *t*-tests, local (*N* = 20): −0.54 ± 0.5, *p* < 0.001; distal (*N* = 19): −0.37 ± 0.78, *p* = 0.053; sham (*N* = 20): −0.58 ± 0.8, *p* < 0.001; verum (*N* = 39): −0.46 ± 0.65, *p* < 0.001]. However, at the three-month follow-up, CTS patients in the local, distal, and combined verum groups showed significant reductions in BCTQ function scores compared to baseline while the sham group did not [paired *t*-tests, local (*N* = 18): −0.39 ± 0.63, *p* = 0.019; distal (*N* = 17): −0.30 ± 0.43, *p* = 0.015; sham (*N* = 16): −0.14 ± 0.6, *p* = 0.37; verum (*N* = 35): −0.35 ± 0.54, *p* < 0.001] (Supplementary Table 4). See Supplementary Materials for more detail.

### Seed-to-Voxel S1 Connectivity During Rest

**Figure 1:**
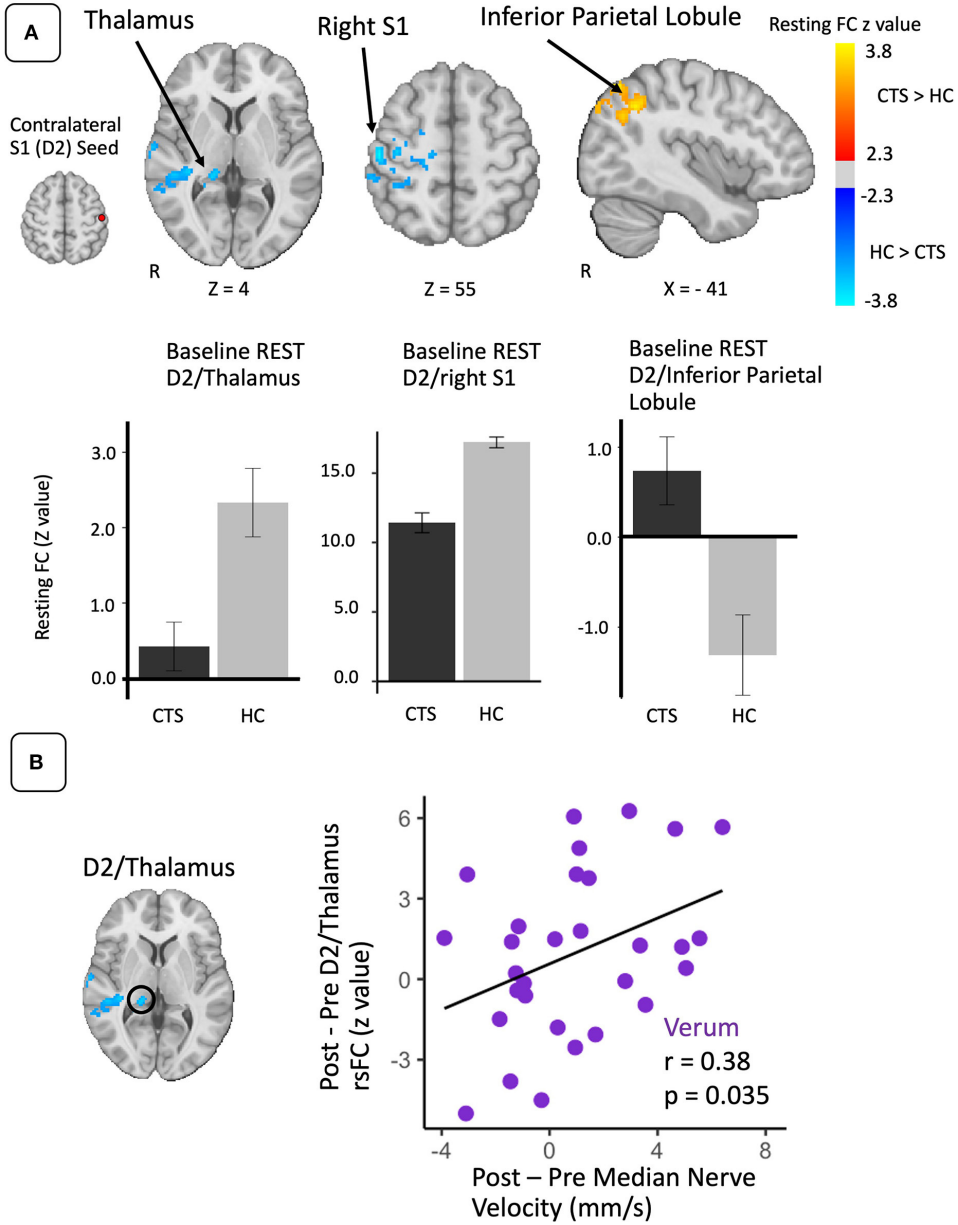
Resting state S1 connectivity is altered in CTS compared to healthy controls and linked with post-therapy changes in peripheral nerve function. **(A)** During REST, compared to healthy controls, the D2 S1 representation (seed) contralateral to more affected hand in CTS was less functionally connected to the right pulvinar nucleus of the thalamus and right (ipsilateral) S1. D2 was also more functionally connected to left inferior parietal lobule in the CTS group. **(B)** Increases in resting D2/thalamus connectivity following verum EA therapy were correlated with post-therapy improvement in median nerve conduction velocity. N.b. S1, primary somatosensory cortex; D2, S1 cortical representation of the second digit of the hand; FC, functional connectivity. ROIs for longitudinal analyses were defined from regions showing differences between CTS patients and healthy controls in resting state D2 functional connectivity at baseline.

As median-nerve innervated D2 has been previously shown to shift relative to D3 in CTS patients (5), we selected the D2 somatosensory representation contralateral to the more affected hand for the seed location and assessed rsfMRI from D2 to the rest of the brain. Compared to HC, CTS patients demonstrated greater D2 functional connectivity to left Inferior Parietal Lobule (IPL) and angular gyrus and lower connectivity to right S1/M1, right pulvinar nucleus of the thalamus, right supramarginal and superior temporal gyri, and right posterior cingulate cortex when compared to HC subjects (Table 1; Figure 1A).

**Table 1:** Resting-state D2 functional connectivity for CTS (*N* = 64) vs. healthy controls (*N* = 29).

	**Side**	**Size (mm^3^)**	**MNI coordinates**			**Peak z-stat**
			**X (mm)**	**Y (mm)**	**Z (mm)**	
**healthy controls > CTS**						
S1	R	13,416	50	−18	55	3.79
Supramarginal gyrus	R	13,416	47	−27	34	3.69
M1	R	13,416	38	−17	55	3.12
Posterior cingulate cortex	R	13,416	13	−23	41	2.79
Superior temporal gyrus	R	8,984	66	−23	9	3.46
Pulvinar nucleus (thalamus)	R	8,984	18	−34	1	3.38
**CTS > healthy controls**						
Inferior parietal lobule	L	4,120	−41	−69	54	3.65
Angular gyrus	L	4,120	−40	−57	39	3.62

Repeated measure ANOVAs with factors of Time (baseline vs. post-therapy) and Treatment (local, distal, or sham) did not find any significant main effects or interactions in resting state functional connectivity values between D2 and any of the ROIs generated from the resting state group difference map (Table 1). However, of these ROIs, only post-therapy change in D2 to right thalamus resting state connectivity for the verum EA group demonstrated a significant correlation to post-therapy change in median nerve conduction velocity (*r* = 0.38, *p* = 0.035, *N* = 31) (Figure 1B). Additional analyses demonstrated that left and right-handed CTS patients displayed no differences in resting state D2 to thalamus functional connectivity (Supplementary Materials).

### Seed-to-Voxel S1 Connectivity During Sustained EA

**Figure 2:**
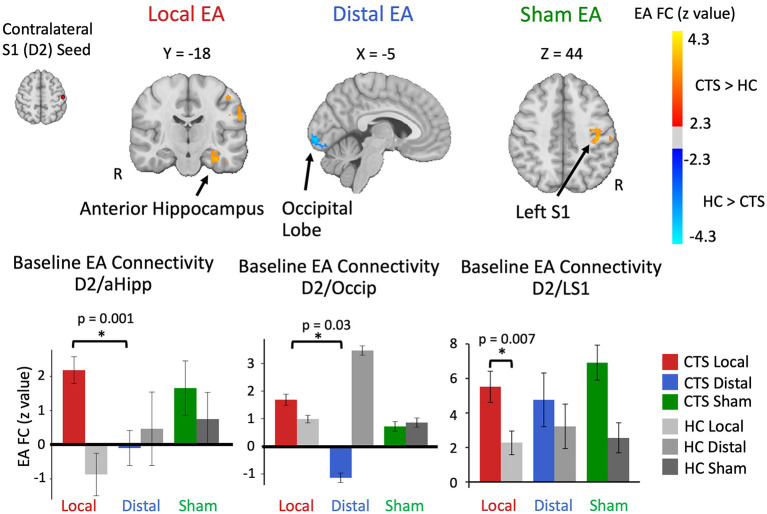
Altered baseline S1 (D2) functional connectivity during sustained electro-acupuncture (EA) in CTS patients. Compared to healthy controls, D2 contralateral to most affected hand in CTS patients was more functionally connected to anterior hippocampus during local EA, less connected to the occipital lobe during distal EA, and more connected to left S1 during sham. Both D2 to anterior hippocampus and D2 to occipital functional connectivity were elevated during local and distal EA for CTS patients in ROI-to-ROI analyses. D2 to left S1 functional connectivity during sustained local EA was elevated for CTS patients compared to healthy controls. HC, healthy controls; S1, primary somatosensory cortex; D2, S1 cortical representation of the second digit of the hand; Occip, occipital cortex; FC, functional connectivity.

D2 to whole brain functional connectivity differences between CTS and HC groups were assessed separately for local, distal, and sham EA (Table 2; Figure 2). During local EA, D2 was more functionally connected to left anterior hippocampus (aHipp), amygdala, inferior insula, parahippocampal gyrus, putamen, and S1 for CTS compared to HC. Of these ROIs, only the D2 to aHipp connectivity showed a significant effect of Treatment Group on within the CTS cohort at baseline (one-way ANOVA, *F* = 4.66, *p* = 0.014). Follow-up testing revealed that D2 to hippocampus connectivity was greater during local compared to distal EA (unpaired *t*-test, *t* = 3.54, *p* = 0.001) in CTS subjects.

**Table 2:** S1 (D2) functional connectivity for CTS patients vs. healthy controls during EA.

	**Side**	**Size (mm^3^)**	**MNI coordinates**			**Peak z-stat**
			**X (mm)**	**Y (mm)**	**Z (mm)**	
**Local EA**						
**CTS** **(***N***=** **22) > HC (***N***= 19)**						
Anterior hippocampus	L	6,168	−30	−14	−24	3.62
Amygdala	L	6,168	−28	−3	18	3.21
Inferior insula	L	6,168	−41	−3	−11	2.69
Parahippocampal gyrus	L	6,168	−31	−39	−8	4.00
Putamen	L	6,168	−31	−11	−9	3.80
Inferior frontal gyrus	L	6,736	−48	12	27	3.66
S1 (BA2)	L	2,800	−45	−36	46	3.40
S1 (BA3b)	L	2,960	−42	−22	46	3.09
**Distal EA**						
**HC (***N***= 16) > CTS (***N***= 19)**						
Occipital lobe	L	4,456	−5	−97	−4	−3.57
**Sham EA**						
**CTS (***N***= 17) > HC (***N***= 12)**						
S1 (BA3a)	L	2,976	−37	−26	42	3.54
Inferior parietal lobule	L	2,976	−52	−28	34	3.08

During distal EA, D2 was less connected to a cluster in the inferior occipital lobe for CTS relative to HC, and the follow-up ROI analysis showed a trend toward significance of Treatment Group on connectivity values (*F* = 2.56, *p* = 0.088) in CTS patients. *Post-hoc* testing revealed lower D2 to occipital lobe connectivity during distal EA compared to local EA for CTS patients (unpaired *t*-test: *t* = −2.25, *p* = 0.03).

During sham EA, D2 was more connected to another S1 subregion in left S1 and the left inferior parietal lobule in CTS patients compared to healthy controls. The left S1 ROI for sham EA also showed significantly higher D2 connectivity for local EA in CTS patients compared to healthy controls (unpaired *t*-test: *t* = 2.86, *p* = 0.007). However, follow-up ANOVA analysis on this ROI found no significant effect of Treatment Group on connectivity values (*F* = 0.80, *p* = 0.46) within CTS patients. Additional analyses demonstrated that left and right-handed CTS patients displayed no differences in D2 to aHipp and D2 to occipital lobe functional connectivity during EA (Supplementary Materials). D2 to left S1 functional connectivity was influenced by Affected Hand of CTS participants, aligned with our S1 subregion analysis (Supplementary Materials).

### Post-therapy Changes in Seed-to-Voxel S1 Connectivity During Sustained EA

**Figure 3:**
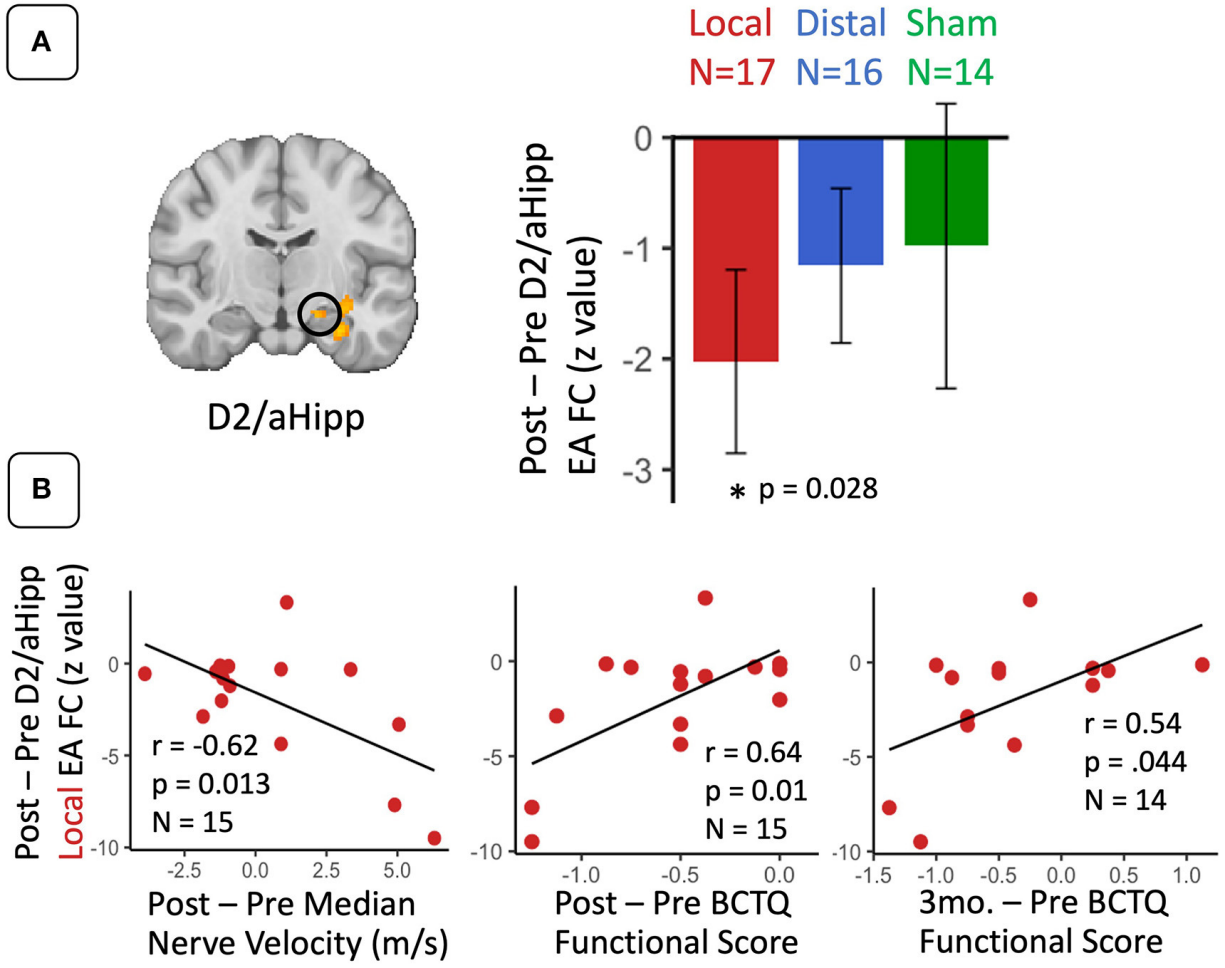
For CTS patients, S1 (D2) functional connectivity to anterior hippocampus during sustained electroacupuncture (EA) were reduced following local EA therapy and linked with clinical variables following local treatment. **(A)** D2 to anterior hippocampus functional connectivity during EA decreased following local treatment only. **(B)** These decreases in D2 to anterior hippocampus connectivity in the local group were correlated with post-therapy changes in median nerve conduction velocity and symptom severity, and 3-month changes in symptom severity. N.B. S1, primary somatosensory cortex; D2, S1 cortical representation of the second digit of the hand; aHipp, anterior hippocampus; FC, functional connectivity. ROIs for this longitudinal analysis were defined from regions showing differences between CTS patients and healthy controls D2 functional connectivity during electro-acupuncture at baseline.

For D2 to aHipp connectivity during EA, a repeated measures ANOVA revealed a main effect of Time (post-therapy vs. baseline) (*F* = 6.85, *p* = 0.013). *Post-hoc* testing demonstrated that D2 to aHipp functional connectivity significantly decreased only for the local EA group (paired *t*-test, *t* = −2.44, *p* = 0.03) (Figure 3A). Furthermore, for the local EA group, change in D2 to aHipp connectivity was negatively correlated with change in median nerve conduction velocity (*r* = −0.62, *p* = 0.013, *N* = 15) and positively correlated with both post-therapy and 3-month follow-up change in BCTQ Functional score (*r* = 0.64, *p* = 0.01, *N* = 15; *r* = 0.54, *p* = 0.044, *N* = 14, respectively) (Figure 3B). Thus, patients in the local EA group showing greater post-therapy decreases in D2 to aHipp connectivity also demonstrated greater improvement in median nerve conduction velocity and greater improvement in functional status. For D2 to occipital lobe connectivity during EA, a repeated measures ANOVA revealed no main effects or Time × Treatment interactions. However, in the distal EA group, post-therapy change in D2 to occipital lobe sustained EA connectivity was positively correlated to post-therapy change in median nerve conduction velocity (*r* = 0.62, *p* = 0.025, *N* = 15). For D2 to left S1 connectivity during EA, a repeated measures ANOVA revealed a trend toward significance for an effect of Time (post therapy vs. baseline, *F* = 3.23, *p* = 0.078). However, follow up paired *t*-tests found no significant post-therapy changes in D2 to left S1 connectivity in any of the treatment groups. Other ROIs did not show significant effects of Time and Treatment Group or correlations to the clinical variables reported above.

## Discussion

Our study applied functional brain connectivity to evaluate CTS neuroplasticity and acupuncture-associated changes for this neuropathic pain disorder. We investigated both resting, or intrinsic, connectivity and state-specific connectivity during EA treatment, revealing dynamic treatment responses that evolve as therapy progresses. We focused our connectivity analyses on the S1 subregion encoding the cortical representation of median-nerve innervated D2, previously shown to exhibit shifted somatotopy in CTS patients (5). D2 connectivity differed between CTS patients and healthy controls not only during resting-state, but also while receiving EA treatment. Furthermore, changes in D2 connectivity during both of these states following therapy were associated with important neurophysiological and symptom-report clinical metrics. Previously, we demonstrated that structural and functional reorganization of S1 occurs in CTS and is responsive to EA therapy (3). Our current investigation extends these findings to identify brain regions, such as the pulvinar nucleus of the thalamus and the anterior hippocampus, whose functional links to S1 are associated with clinically-relevant response to a multi-week course of EA therapy. Our findings reveal a more expanded brain plasticity in CTS, beyond S1, and further our understanding of how EA directed to local or distal body regions, relative to the CTS-affected wrist, may lead to improvements in symptoms and peripheral median nerve dysfunction.

Functional brain connectivity is most commonly applied to data collected during a minimally-controlled “resting” state, which reflects both trait and state-like properties of brain connectivity (29). In this study, we investigated S1 functional connectivity during resting state and while subjects received sustained local, distal, or sham EA. During this more controlled state, the anterior hippocampus emerged as a region whose functional coupling to the D2 representation in S1 during local EA (but not distal or sham EA) was associated with clinical response to EA therapy. Specifically, relative to HC, CTS patients showed increased D2/anterior hippocampus connectivity during local EA, which was reduced following a longitudinal course of local EA therapy. Thus, in addition to S1 somatotopic re-organization (3), our results demonstrate that S1 connectivity to subcortical structures can also change following a course of acupuncture therapy. Additionally, we found that the change in S1/hippocampus local EA connectivity following therapy was associated with recovery of peripheral nerve health and patients' functional abilities. This result underscores the importance of assessing functional connectivity during the EA therapy itself to evaluate potential mechanisms of action for post-therapy improvements in clinical outcomes. The relevance of the hippocampus in pain processing has been previously reported in several contexts. In chronic low back pain, resting S1/hippocampus connectivity was linked to pain severity (30). During evoked pain, hippocampus activation is sensitive to expectations created by placebo and nocebo contexts (31). While memory-related functions are supported by dorsal hippocampus, emotion and affect regulation have been linked to ventral and anterior hippocampal regions (32), which we found to be linked with S1 in this study. Furthermore, functional response to pain in the hippocampus has been shown to mediate the association between pain and basal cortisol levels (33), suggesting a link between this region and hormonal/autonomic response to pain. In fact, tracing research in animal models has uncovered multi-synaptic pathways linking the hippocampus with brainstem nuclei involved in autonomic regulation (34), and a human neuroimaging meta-analysis localized the hippocampus within the central autonomic network (35). Thus, changes in S1/hippocampus connectivity during local EA might also be associated with peripheral nerve function and behavioral improvements via autonomic regulation of local blood flow to maintain peripheral nerve health (36) at the wrist.

While EA applied locally to the site of neuropathy has the added benefit of direct impact to vascular flow at the needle (37), and potentially median nerve vasa nervorum (38), distal EA also showed improvements in nerve function and symptoms, suggesting indirect, central nervous system-mediated pathways for benefiting CTS (3). During sustained distal EA, CTS patients demonstrated decreased connectivity between D2 and occipital lobe compared to HC, and greater increase following distal EA therapy was associated with greater improvement in peripheral nerve health. While the occipital lobe is not as commonly implicated in chronic pain, primary visual processing and visual network resting connectivity was found to be altered in chronic low back pain (39) and correlated with chronic pain duration. Furthermore, nociceptive processing has been shown to extend outside of typical somatosensory and limbic areas to include primary visual areas of the occipital lobe (40). Thus, increased somatosensory/visual coupling in the occipital lobe during distal EA may reflect normalization of median nerve afference and improved peripheral nerve health following distal EA for CTS.

Resting S1 (D2) connectivity was also altered in CTS. Compared to HC, we found reduced resting D2 connectivity to the right pulvinar nucleus of the thalamus. Although EA therapy did not restore resting state D2 to thalamus connectivity to healthy control levels, greater increases in S1/pulvinar resting state connectivity following verum EA were associated with greater recovery of peripheral nerve function (i.e., increased median nerve velocity). A study applying motor cortex transcranial direct current stimulation therapy to fibromyalgia patients similarly did not find significant changes in S1 to thalamus functional connectivity, even though changes in connectivity were linked to pain analgesia (41). It's possible that these S1 to thalamus functional relationships adapt and respond to therapy over longer timescales in chronic pain conditions, potentially reflecting affective and cognitive, rather than somatosensory, processes supported by S1 to pulvinar connectivity. A recent functional parcellation of the thalamus places the cluster identified in our analysis predominantly in the ventromedial pulvinar nucleus (42). A Neurosynth meta-analytic topic map (43) suggests that this ROI has also been found to be activated in prior fMRI studies in emotion and pain topic areas (42). The ventromedial pulvinar is largely composed of matrix cells which have diffuse but widely distributed connections to the cortex (44). In contrast to the more feedforward sensory regions of the thalamus featuring core cells, which, for example, transfer somatosensory information from the periphery to S1, matrix cell regions of the thalamus are more involved with associative processing and have preferential functional connectivity with default, control, limbic, and ventral attention networks (44). Additionally, cortical connections with matrix cell thalamic regions show more variable functional dynamics, and longer timescales of intrinsic (resting) connectivity, suggesting a more modulatory role (44). Thus, reduced S1/pulvinar connectivity in CTS patients may result from somatotopic neuroplasticity in S1 combined with altered pain and affective processing. Aberrant S1/pulvinar resting connectivity has been noted in previous pain-related studies. We recently showed that acutely increasing low back pain in chronic low back pain patients increased connectivity between the S1 cortical representation of the low back and dorsal/anterior regions of the thalamus, partially overlapping the pulvinar nucleus (45). Taken together, our results suggest that altered S1/pulvinar connectivity in CTS reflects ongoing and intrinsic neuromodulatory processes reflecting cognitive/affective dimensions of clinical pain, rather than nociception, and is responsive to verum EA therapy.

We have previously showed that a longitudinal course of EA therapy can improve maladaptive structural and functional S1 plasticity in CTS (3). However, it is not entirely clear how the ameliorating effects of EA therapy are transduced from peripheral stimulation to brain plasticity. Interestingly, relative to healthy controls, CTS patients showed greater connectivity within S1 during *both* local and sham EA (Figure 2; Supplementary Figure 3). While sham EA did not involve needle insertion or electrical current, subjects randomized to sham EA were instructed to focus attention on the (non-penetrating) needles located near the wrist. Thus, attending to the wrist may have increased awareness of pain/paresthesia sensations from this region for CTS patients, potentially reflected in greater within S1 connectivity during sham EA. Indeed mental imagery of finger vibrotactile stimulation has been shown to elicit specific S1 activation that overlaps partially with that of actual stimulation (46, 47). However, functional connectivity of S1 to several cortical regions was notably different between real and imagined stimulation (46). Ultimately, functional connectivity within S1 may be similarly manipulated by real and sham EA for cortical representations of body regions local to the neuropathy, while S1 connectivity to other cortical/subcortical regions may underlie broader top-down mechanisms for improved peripheral nerve function.

Several limitations should be noted. Our study employed a longitudinal, randomized design to evaluate the efficacy of three different EA therapies. While the treatment sessions used a hybrid paradigm that combined manual and electro-acupuncture, brain imaging data were collected during EA and not manual needle stimulation. Thus, exploring differences in brain connectivity between manual acupuncture and EA, or which method contributes more to clinical improvement, is beyond the scope of this work. Participant dropout and removal of low-quality data ultimately reduced the sample size for each treatment arm, affected the power of statistical analyses and generalizability of the results. Additionally, we should note that our approach was unable to determine whether any of the changes in functional connectivity patterns were causally driving the clinical/behavioral improvements observed, or were downstream biomarkers of improvements in peripheral nerve function.

In summary, we have demonstrated that CTS pathology and EA therapy affect functional connectivity not only within S1 regions, but also between S1 and subcortical regions such as the pulvinar and anterior hippocampus. Furthermore, we provide evidence that assessing functional connectivity not just during rest but also during EA application can reveal novel insights into brain-focused mechanisms supporting peripheral neuromodulation therapy.

## Data Availability Statement

The raw data supporting the conclusions of this article will be made available by the authors, without undue reservation.

## Ethics Statement

The studies involving human participants were reviewed and approved by Massachusetts General Hospital and Partners Human Research Committee. The patients/participants provided their written informed consent to participate in this study.

## Author Contributions

VN, NK, and JA contributed to conception and design of the study. RS, YM, JK, CM, and JG executed the study, collected, and organized data. HF performed the statistical analysis. HF and VN wrote the manuscript. All authors contributed to manuscript revision, read, and approved the submitted version.

## Funding

This work was generously supported by the NIH (National Center for Complementary and Integrative Health grants R01-AT004714, R01-AT007550, R61/R33-AT009306, and P01-AT009965 and National Center for Research Resources grants P41-RR14075, S10-RR021110, and S10-RR023043) and Korea Institute of Oriental Medicine (grant number KSN2021240).

## Conflict of Interest

The authors declare that the research was conducted in the absence of any commercial or financial relationships that could be construed as a potential conflict of interest.

## Publisher's Note

All claims expressed in this article are solely those of the authors and do not necessarily represent those of their affiliated organizations, or those of the publisher, the editors and the reviewers. Any product that may be evaluated in this article, or claim that may be made by its manufacturer, is not guaranteed or endorsed by the publisher.
