# Supplementary material for: S1 Brain Connectivity in Carpal Tunnel Syndrome Underlies Median Nerve and Functional Improvement Following Electro-Acupuncture
Source: Front Neurol. 2021 Oct 27;12:754670. doi: 10.3389/fneur.2021.754670 (PMC8578723; doi:10.3389/fneur.2021.754670)
Supplement: Supplementary file 1 [file Data_Sheet_1.pdf]

Supplementary Material

S.1 BOLD fMRI Data Pre-processing and Analysis Workflow

BOLD fMRI data were preprocessed to remove sources of noise before performing functional connectivity analyses between S1 subregions and between the second hand digit S1 representation (D2) and the rest of the brain (**Supplementary Figure 1**). Detailed reporting on the number of scans that passed quality control for use in functional connectivity analyses is found in **Supplementary Table 1.**

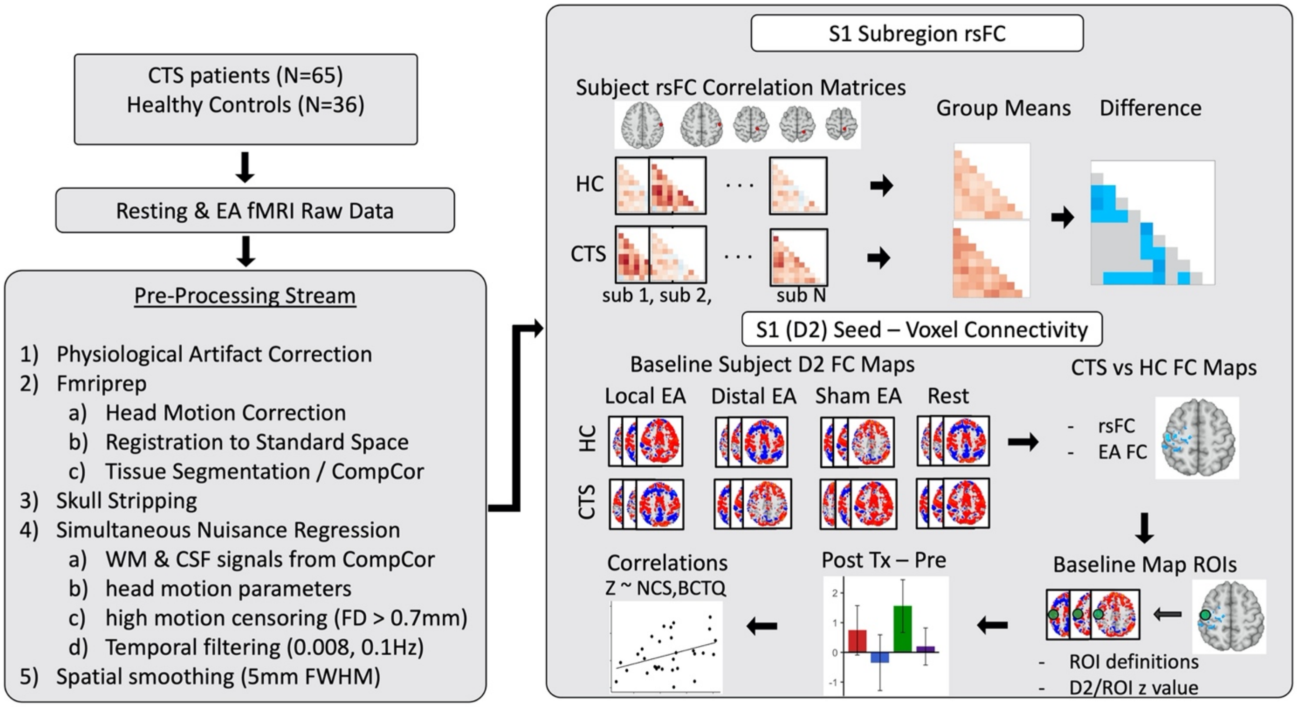

Supplementary Figure 1. BOLD fMRI data pre-processing and analysis workflow.

The BOLD scans with sufficient quality are reported in **Supplementary Table 1**, where the number of EA scans represents having at least one EA scan (either EA1, EA2, or both) passing the quality assessments.

Supplementary Table 1.
Distribution of BOLD data with sufficient quality across groups

		Local		Distal		Sham	
		**REST**	**EA**	**REST**	**EA**	**REST**	**EA**
**HC**	Baseline	21	19	18	16	14	12
**CTS**	Baseline	20	22	20	19	20	17
	Post-Therapy	18	17	17	16	17	14

S.2 Demographic and clinical characterization

CTS and HC groups did not differ in age or sex, nor were there differences between the different EA therapy groups within the CTS cohort (**Supplementary Table 2**). Proportions of affected hand did not differ between treatment groups for CTS patients (χ^2(df =2, N= 65) = 0.93, p = 0.63, **Supplementary Table 2**). As expected, CTS patients had lower median nerve conduction velocity compared to healthy controls (**Supplementary Table 3**). After longitudinal EA therapy, only the verum group (local+distal subjects) showed significant increases in nerve conduction velocity. CTS subjects were also assessed for functional abilities with the BCTQ at baseline, post-therapy, and at a 3 month follow-up (**Supplementary Table 4)**. Compared to baseline, the local, sham, and verum groups experienced significant reductions in BCTQ function scores at the post-therapy timepoint. At the 3 month follow-up, the local, distal, and verum groups showed significantly decreased BCTQ function scores while the sham group did not.

Supplementary Table 2: Demographic Data

	N Males	N Females	# Left Hand Affected	Mean Age
**HC**	10	19	n/a	47.86 +/- 9.19 yrs
**CTS**	10	54	23	49.11 +/- 8.97 yrs
**Local**	4	18	8	48.59 +/- 10.31 yrs
**Distal**	4	17	6	48.43 +/- 8.66 yrs
**Sham**	2	19	9	50.33 +/- 8.03 yrs

Supplementary Table 3: Average D2,D3 Median Nerve Velocity

	Baseline (m/s)	Post (m/s)	N	Post – Baseline (m/s)	t-value	p-value
**HC**	53.61 +/- 5.50		29			
**CTS**						
**Local**	37.97 +/- 6.23	38.63 +/- 6.13	20	0.67 +/- 2.66	1.17	0.28
**Distal**	36.63 +/- 7.71	37.88 +/- 7.42	18	1.24 +/- 2.78	1.90	0.074
**Sham**	38.70 +/- 9.07	38.32 +/- 8.93	21	-0.38 +/- 3.57	-0.49	0.63
**Verum**	37.34 +/- 6.90	38.28 +/- 6.53	38	0.94 +/- 2.69	**2.15**	**0.038**

Supplementary Table 4: Boston Carpal Tunnel Syndrome Questionnaire Function Scores

	Local (N=20)	Distal (N=19)	Sham (N=20)	Verum (N=39)
Baseline Score	2.08 +/- 0.72	2.11 +/- 0.75	2.05 +/- 0.70	2.07 +/- 0.72
Post Score	1.54 +/- 0.50	1.74 +/- 0.78	1.47 +/- 0.80	1.65 +/- 0.69
Post-Baseline	-0.54 +/- 0.50	-0.37 +/- 0.78	-0.58 +/- 0.80	-0.46 +/- 0.65
t-value	-4.86	-2.07	-3.26	-4.42
p-value	**< 0.001**	0.053	**< 0.001**	**< 0.001**
3 mo. N	18	17	16	35
3 Month Score	1.69 +/- 0.63	1.75 +/- 0.40	1.91 +/- 0.60	1.74 +/- 0.54
3mo-Baseline	-0.39 +/- 0.63	-0.30 +/- 0.43	-0.14 +/- 0.60	-0.35 +/- 0.54
t-value	-2.61	-2.77	-0.93	-3.71
p-value	**0.019**	**0.015**	0.37	**< 0.001**

S.3 Baseline S1 subregion seed-to-seed resting state functional connectivity analysis

Average timeseries were extracted from the following somatotopic specific regions of S1: second digit of the hand (D2), back, chest, face, and leg. The MNI space localization were taken from prior research by our own group and others (see **Supplementary Table 5** for seed coordinates and source) using 3mm radius seeds (Nilearn, (Abraham et al., 2014)) from the denoised REST and sustained EA scans. Seeds were placed both contralateral and ipsilateral to the more affected hand (dominant hand for HCs) for each subject. The timeseries were variance normalized (dividing by the standard deviation of the timeseries), and then correlated pairwise to generate a correlation matrix, which was then transformed with Fisher's r-to-z (inverse hyperbolic tangent). Transformed correlation matrices from repeated fMRI scans were averaged together for EA scans where applicable. The transformed correlation matrices were compared between HC and CTS groups at baseline using two-sample, unpaired T-tests on each square of the lower triangle (excluding the diagonal), correcting for multiple comparisons using False Discovery Rate (FDR). A one-way ANOVA (factor Treatment: local, distal, or sham) was performed within the CTS group for each ROI-ROI connection.

At baseline, CTS and HC groups showed positive ROI-to-ROI correlations within the S1 connectivity matrices (**Supplementary Figure 2**). When contrasting CTS and HC groups, the S1 ROI connectivity matrices showed lower functional connectivity between S1 nodes in CTS compared to HC, both for intra and inter-hemispheric connections. Specifically, four connections within contralateral S1, and two connections between contralateral and ipsilateral S1 all showed lower connectivity in CTS patients compared to HCs. There were no differences between CTS and HC groups for S1 functional connectivity during any of the EA types, and there was no significant effect of EA type within the CTS group.

Whole-brain resting state functional connectivity of the D2 subregion of S1 also showed decreased connectivity to other S1 regions, similar to a previous report in CTS patients (Lu et al., 2017). We further explored resting connectivity within S1 with a ROI approach. Compared to HC, CTS patients demonstrated reduced resting connectivity between several S1 subregions. Interestingly, reduced connectivity within S1 is consistent with our prior study in fibromyalgia patients (Kim et al., 2015), a chronic widespread (Ellingsen et al., 2020) pain disorder. While CTS patients displayed reduced connectivity for fewer S1 subregions compared to fibromyalgia, non-hand

regions also showed altered functional connectivity in CTS, indicating that even for a relatively circumscribed neuropathic pain disorder like CTS, S1 plasticity in terms of functional connectivity is not entirely restricted to cortical representations of the site of pain. While we did not evaluate pain widespreadness in CTS patients for this study, the broadening of CTS-associated S1 plasticity suggests potential mechanisms through which non-local (i.e. distal) acupuncture may also benefit CTS.

Supplementary Table 5.
Somatosensory cortex subregions used for resting state functional connectivity analysis

Seed Name	MNI Coordinates			Publication Source
	X (mm)	Y (mm)	Z (mm)	
Finger D2	+/-51	-18	54	(Napadow et al., 2007)
Back	+/-18	-44	64	(Lloyd et al., 2008)
Chest	+/-18	-36	64	(Strigo et al., 2003)
Face	+/-60	-14	40	(Moulton et al., 2009)
Leg	+/-8	-38	68	(Kim et al., 2015)

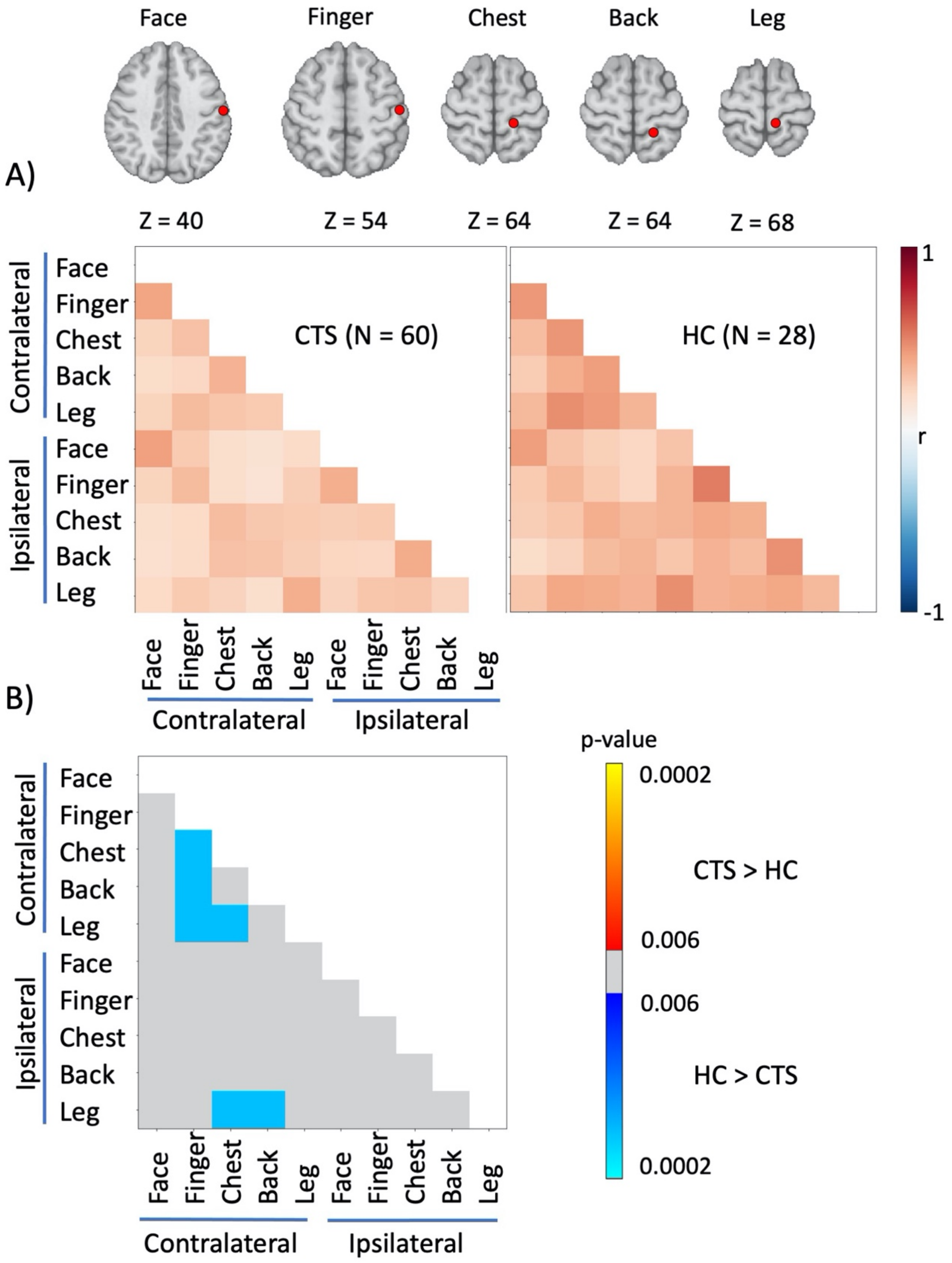

Supplementary Figure 2. CTS patients display lower resting state functional connectivity between several S1 subregion compared to healthy controls. (A) Both CTS and healthy control (HC) subjects display positive correlations between S1 subregion BOLD fMRI signals during rest. (B) Several inter-region correlations between somatotopic cortical representations contralateral and ipsilateral to the more affected hand during rest were lower for CTS compared to HC groups. S1: primary somatosensory cortex.

S.4 CTS>HC similarity in D2 connectivity during local and sham sustained EA

Relative to healthy controls (HC), CTS patients display higher functional connectivity between left D2 and surrounding left S1 areas during both local and sham sustained EA. More specifically, there is an overlap of 111 voxels (888mm^3)

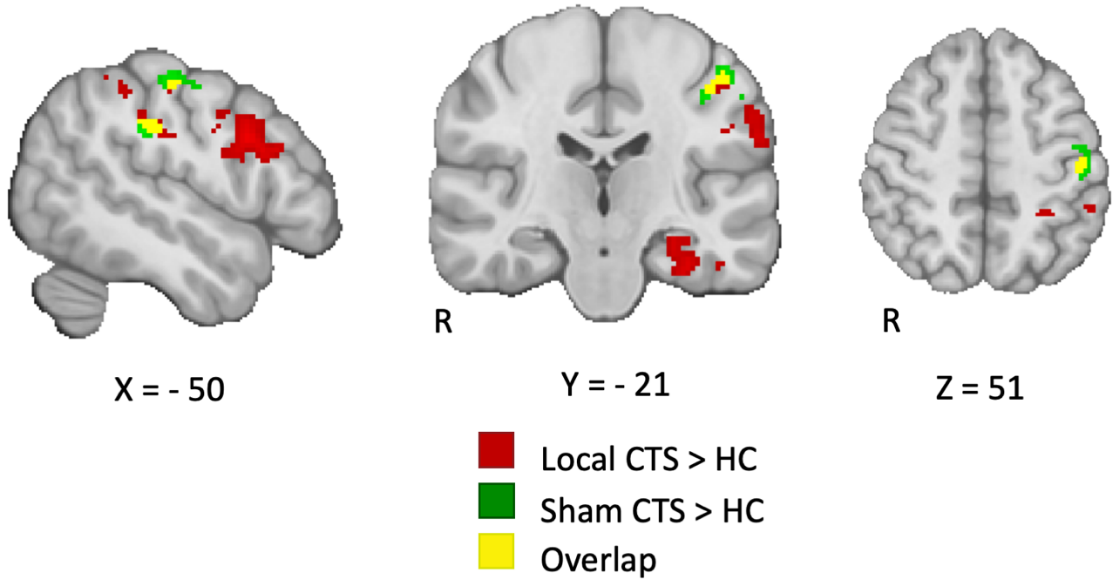

Supplementary Figure 3. Overlap (yellow) between increased CTS (vs HC) S1 (D2) functional connectivity during local (red) and sham (green) sustained electro-acupuncture (EA). Regions that show greater functional connectivity to left D2 during local and sham EA overlap at 111 voxels (888 mm^3), comprising left the primary somatosensory cortex (S1) and the left anterior supramarginal gyrus (SMG). HC: healthy controls; S1: primary somatosensory cortex; D2: S1 cortical representation of the second digit of the hand.

S.5 Affected hand of CTS patients influences S1 connectivity within S1, but not to other brain regions

For the analysis of S1 functional connectivity, we extracted timeseries from the D2 seed contralateral to the more affected hand for each CTS patient. In order to maintain any lateralized functional brain specificity across right- and left-hand affected CTS, whole brain BOLD fMRI data were *not* flipped. As an example, the right pulvinar thalamus region identified in the CTS vs HC contrast in the baseline REST data was contralateral to the D2 seed for right-hand affected CTS patients, and ipsilateral to the D2 seed for left-hand affected CTS patients. We ran post-hoc tests to verify that D2 functional connectivity to brain regions of interest identified in main analysis results was not strongly influenced by D2 seed laterality. We found that none of the main results showed significant effects of left versus right D2 seed laterality. Unfortunately, the low numbers of left-hand CTS subjects in each EA treatment group made several comparisons too underpowered to be considered reliable (especially baseline vs post-therapy comparisons).

Resting state functional connectivity between D2 and the right pulvinar subregion of the thalamus ROI was not significantly different between left- and right-hand affected CTS patients at baseline (right hand: 0.15 +/- 2.52 z-value, N=39; left-hand 1.06 +/- 2.44, N=20; t = -1.34, p = 0.2). The D2 connectivity to this thalamus ROI remained significantly lower in CTS patients compared HC subjects when including only right-hand affected CTS subjects (t = -3.35, p = 0.002) and trending toward significance when including only left-hand affected CTS subjects (t = -2.02, p = 0.05). Furthermore, there were no significant differences in the change in D2 to thalamus connectivity between left- and right-hand affected CTS subjects that received verum EA therapy (right-hand: 1.07 +/- 2.89 z-value, N = 23; left-hand: 0.49 +/- 3.40, N = 9; t = 0.45, p = 0.66).

An ANOVA on baseline functional connectivity between D2 and the left anterior hippocampus ROI during sustained EA showed a significant main effect of Treatment Group (F = 4.77, p = 0.013), but not Affected Hand (F = 0.094, p = 0.76) in CTS subjects. Further testing revealed no significant differences in baseline D2 to anterior hippocampus connectivity during EA between left- and right-hand affected CTS subjects in the local treatment group (right-hand: 2.64 +/- 1.86 z-value, N=14; left-hand: 1.26 +/- 1.31, N=7; t = 1.43, p = 0.20). Additionally, D2 connectivity to this anterior hippocampus ROI remained significantly higher in CTS patients compared to HC subjects during sustained local EA when considering only right-hand affected patients (t = 3.16, p = 0.004). The post-therapy decrease in D2 to anterior hippocampus functional connectivity also remained significant when including only right-hand affected CTS subjects (-2.95, p = 0.015, N = 11).

An ANOVA on baseline functional connectivity between D2 and the occipital lobe ROI during sustained EA showed neither a significant main effect of Treatment Group (F = 2.42, p = 0.1), nor of Affected Hand (F = 0.23, p = 0.6) in CTS subjects.

An ANOVA on baseline functional connectivity between D2 and the left S1 ROI during sustained EA showed a strong main effect of Affected Hand (F = 15.23, p < 0.001), but not Treatment Group (F = 1.04, p = 0.36) in CTS subjects. Further testing revealed a significant lower baseline D2 to left S1 connectivity during EA in right-hand compared to left-hand affected CTS subjects (right-hand: 3.98 +/- 4.30 z-value, N = 34; left-hand: 8.91 +/- 4.10, N = 18; t = -3.09, p = 0.003). This result indicates that intra-hemisphere S1 connectivity (left D2 seed to left S1 for right-hand affected CTS) was lower than inter-hemisphere S1 connectivity (right D2 seed to left S1 for left-hand affected CTS) during sustained EA, in accordance with our analysis of S1 subregion connectivity during resting-state reported above (**Supplementary Figure 2)**. The higher proportion of right-hand affected subjects may have led to the significant cluster present in left, but not right, S1 for the CTS vs. HC group contrast map. Overall, connectivity within S1 was sensitive to D2 seed laterality. However, D2 seed laterality did not affect connectivity to non-somatotopic areas in the rest of the brain.
